# Bipartite Fault-Tolerant Consensus Control for Multi-Agent Systems with a Leader of Unknown Input Under a Signed Digraph

**DOI:** 10.3390/s25051556

**Published:** 2025-03-03

**Authors:** Anning Liu, Wenli Zhang, Dongdong Yue, Chuang Chen, Jiantao Shi

**Affiliations:** College of Electrical Engineering and Control Science, Nanjing Tech University, Nanjing 211816, China

**Keywords:** bipartite consensus, fault-tolerant control, multi-agent system (MAS), intermediate estimator

## Abstract

This paper addresses the bipartite consensus problem of signed directed multi-agent systems (MASs) subject to actuator faults. This problem plays a crucial role in various real-world systems where agents exhibit both cooperative and competitive interactions, such as autonomous vehicle fleets, smart grids, and robotic networks. To address this, unlike most existing works, an intermediate observer is designed using newly introduced intermediate variables, enabling simultaneous estimation of both agent states and faults. Furthermore, a distributed adaptive observer is developed to help followers estimate the leader’s state, overcoming limitations of prior bounded-input assumptions. Finally, simulation results demonstrate the method’s effectiveness, showing that consensus tracking errors converge to zero under under various fault scenarios and input uncertainties.

## 1. Introduction

In recent years, with the advancement of micro-control and wireless communication technologies, the cooperative control of multi-agent systems has attracted extensive attention [1,2,3]. Due to their ability to accomplish complex tasks that a single agent cannot achieve, multi-agent systems have been widely applied in fields such as formation flight [4], smart grids [5], and synchronization of robotic arms [6]. As a core subject in cooperative control, consensus aims to achieve uniformity in states such as position and velocity, and has been extensively studied [7,8,9].

For traditional consensus problems in multi-agent systems, most existing studies primarily focus on cooperative relationships among neighboring agents [10,11,12,13], where the edge weights in the communication topology are non-negative. This cooperative framework, however, is limited to achieving a single collaborative task. In contrast, agents may also exhibit competitive relationships due to conflicts, enabling the accomplishment of collaborative tasks with multiple sub-goals under a local adversarial framework. In practice, many network topologies involve both cooperative and adversarial interactions among agents, which can typically be modeled using signed graphs. Research on such behaviors has practical applications in biological systems [14], trust-distrust social networks [15], opinion dynamics [16], and so on.

During the past years, bipartite consensus, derived from traditional consensus [17,18,19,20], has emerged as a key concept, aiming to design distributed controllers that allow two groups of agents with competitive relationships to asymptotically converge to equal-magnitude but opposite-sign final values. For example, the reference [21] provides sufficient conditions for achieving bipartite state consensus based on an adaptive event-triggered strategy. The reference [22] addresses bipartite state consensus for heterogeneous nonlinear dynamics. The reference [23] investigates bipartite consensus control laws based on distributed disturbance observers, resolving the bipartite consensus tracking problem for nonlinear multi-agent systems with time delays. However, the aforementioned studies do not account for the occurrence of faults in multi-agent systems, leaving this critical aspect unexplored.

The bipartite fault-tolerant cooperative control of multi-agent systems (MASs) plays a crucial role in various real-world applications. For instance, in autonomous vehicle fleets, vehicles must cooperate to maintain formation while also handling competitive interactions, such as lane changes, where actuator faults (e.g., steering or braking failures) can significantly impact stability. Generally, these systems function under high-load conditions, where faults become unavoidable, including actuator failures [24], component malfunctions [25], and sensor issues [26]. These faults can impact the performance and even the stability of subsystems. Considering both competitive relationships and complex fault types further complicates controller design. For instance, the reference [27] addresses the bipartite fault-tolerant state consensus problem under actuator faults by employing event-triggered and output feedback strategies. However, the results are limited to undirected communication topologies and cannot be extended to more general directed communication topologies. The reference [28] advances this work by designing a distributed bipartite output consensus control algorithm for component faults and signed directed graphs. It is worth noting that this study focuses on autonomous discrete-time multi-agent systems with no input signals from the leader.

Moreover, for leader–follower multi-agent systems, the input signal of the leader is inevitable, as it not only affects the leader’s own behavior but also indirectly regulates the dynamics of the entire system through its influence on the followers. In practical systems, the input signal is often unknown and time-varying [29], posing a significant challenge. Some researchers have addressed this challenge by assuming that the input signals are limited [30,31,32]. The reference [33] addresses a class of multi-input multi-output (MIMO) linear multi-agent systems with nonzero input signals under switching topologies. Similarly, the reference [34] investigates the vortex formation control of linear multi-agent systems with unknown leader inputs and external disturbances. However, these studies typically rely on the assumption that the leader’s nonzero input signal is bounded. This bounded input signal assumption is conservative and reduces the applicability of the results to real-world scenarios.

According to the above analysis, we address the bipartite fault-tolerant consensus problem in continuous-time multi-agent systems with actuator faults and a signed directed graph, considering an unknown leader input. Unlike existing methods, our approach enhances robustness by eliminating strict observer matching conditions, making it more adaptable to diverse actuator faults and input uncertainties. Additionally, it accounts for both cooperative and competitive interactions among agents, a challenge that has received limited attention in prior research. The primary contributions of our research are as follows:

(1) Most existing studies on the consensus control problem of MASs primarily consider unsigned graphs [7,8,9,10,11,12]. In contrast, this paper broadens the scope by analyzing directed networks that include both positive and negative weights. Furthermore, unlike the studies in [17,18,19,20], which achieve bipartite consensus without considering actuator faults, we address the bipartite consensus problem under actuator faults. This consideration aligns more closely with practical scenarios and enhances the robustness of MASs.
(2) Due to the leader’s unknown input signal, the follower agents cannot acquire precise information regarding the leader. In contrast to the common assumption of bounded unknown input signals [30,31,32], this paper examines the unknown input signal and employs parameterization theory to design a distributed leader-state observer. This approach allows follower agents to effectively track the leader’s signal.
(3) This paper designs intermediate observers using auxiliary variables to estimate follower states and time-varying actuator faults simultaneously. Drawing from these observers, a bipartite fault-tolerant consensus control protocol is proposed. Unlike existing studies, this approach eliminates the observer matching condition, significantly broadening its applicability.

The rest of this article is structured as follows: Section 2 outlines the problem statement for the bipartite fault-tolerant consensus control in the studied multi-agent system. Section 3 details the design of the state observer, fault estimator, fault-tolerant control law, and the main theoretical findings. Section 4 presents simulation results to validate the proposed approach. Lastly, Section 5 concludes the article.

*Notations:* The notations employed in this article follow standard conventions. Specifically, R represents the set of real numbers, while $R^{n}$ denotes the set of vectors with appropriate dimensions. The expression $\left\{\cdot \right\}$ indicates a block-diagonal matrix. For a matrix *X*, $X^{T}$ denotes its transpose. $I_{N}$ denotes the *N*-dimensional identify matrix, and $sgn\left(\cdot \right)$ represents signal function. ⊗ represents the Kronecker product. The maximal eigenvalues of matrix *Q* is defined as $\lambda_{max}\left(Q\right)$.

## 2. Preliminaries and Problem Description

### 2.1. Graph Theory

Let G(V,E,A) represent a directed graph, where $V=\{v_{1},v_{2},\ldots ,v_{N}\}$ is the set of *N* nodes, each corresponding to an agent. The edge set E⊆V×V defines the communication links between nodes, where $(v_{j},v_{i})\in E$ indicates that node $v_{j}$ can send information to node $v_{i}$. Based on this, the neighbor set of node $v_{i}$ is given by $N_{i}=\{v_{j}\in V|\left(v_{j},v_{i}\right)\in E,\;j\neq i.$ The G’s adjacency matrix is denoted as $A=\left[a_{ij}\right]\in R^{N\times N}$, where $a_{ij}>0$ if and only if $(v_{j},v_{i})\in E,$ and $a_{ij}=0$ otherwise. $a_{ij}>0$ indicates a cooperative relationship between agents, while $a_{ij}<0$ represents a competitive one. Define $\bar{D}=\mathrm{diag}\left({\bar{d}}_{i}\right)\in R^{N\times N}$ as the degree matrix of G, where ${\bar{d}}_{i}=\sum_{j\in N_{i}}a_{ij}$. The Laplacian matrix of the graph G is then given by $L=\bar{D}-A=\left[l_{ij}\right]\in R^{N\times N}$, where $l_{ii}=\sum_{j\in N_{i}}\left|a_{ij}\right|,\;l_{ij}=-a_{ij}\;\mathrm{for}\;i\neq j$. Additionally, let $v_{0}$ denote the leader node. To describe the interactions between the leader and followers, introduce the diagonal matrix $G=\mathrm{diag}\left(g_{1},g_{2},\ldots ,g_{N}\right)\in R^{N\times N}$. If node $v_{i}$ can receive information from the leader $v_{0}$, then $g_{i}>0$; otherwise, $g_{i}=0$.

**Definition** **1** ([35])**.** *If the set of nodes V is partitioned into two subsets, $V_{p}$ and $V_{q}$, the graph G is considered structurally balanced, where $V_{p}\cup V_{q}=V$ and $V_{p}\cap V_{q}=⌀$. This implies the following conditions:*

$$\left\{\begin{array}{cc} a_{ij}\geq 0, & \;\forall v_{i},v_{j}\in V_{p}\;\mathrm{or}\;v_{i},v_{j}\in V_{q},\\ a_{ij}\leq 0, & \;\forall v_{i}\in V_{p},\;v_{j}\in V_{q}. \end{array}\right.$$

*Additionally, a symbol matrix $D=\mathrm{diag}(d_{i})$ is defined, where*

$$d_{i}=\left\{\begin{array}{cc} 1, & \mathrm{if}\;v_{i}\in V_{p},\\ -1, & \mathrm{if}\;v_{i}\in V_{q}. \end{array}\right.$$

For a signed directed graph G that meets the condition in Definition 1, define $L_{G}=L+G$.

In the following, we will introduce the notation for signed digraphs and elaborate on the relationship between the Laplacian matrix and signed graphs. A signed digraph G=(V,E,A) consists of a set of nodes $V=v_{1},v_{2},\ldots ,v_{N}$, a set of directed edges E∈V×V, and a signed adjacency matrix $A=[a_{ij}]$, where $a_{ij}$ represents the weight of the edge from node $v_{j}$ to node $v_{i}$. Unlike standard graphs, the edge weights in a signed digraph can be positive (cooperative interaction) or negative (competitive interaction):

$$\left\{\begin{array}{cc} a_{ij}>0, & if\;v_{j}\;cooperates\;with\;v_{i},\\ a_{ij}<0, & if\;v_{j}\;competes\;with\;v_{i},\\ a_{ij}=0, & if\;there\;is\;no\;direct\;interaction. \end{array}\right.$$

The Laplacian matrix of a signed digraph is given by L=D−A, where *D* is the degree matrix, a diagonal matrix with elements $d_{ii}=\Sigma_{j\neq i}\left|a_{ij}\right|$.

### 2.2. Problem Description

In this article, the model of the ith(i=1,⋯,N) agent is

(1) $$\left\{\begin{array}{c} {\dot{x}}_{i}\left(t\right)=Ax_{i}\left(t\right)+Bu_{i}\left(t\right)+Ef_{i}\left(t\right)\\ y_{i}\left(t\right)=Cx_{i}\left(t\right) \end{array}\right.$$

where $x_{i}\left(t\right)\in R^{n}$ is the state; $u_{i}\left(t\right)\in R^{p}$ is the control input; $f_{i}\left(t\right)\in R^{q}$ is the fault vector; $y_{i}\left(t\right)\in R^{m}$ is the measurement; A,B,C,E are known matrices with appropriate dimensions, and the pair (A,B) is both controllable and detectable.

The dynamics of the leader agent, labeled as 0, are described by the following equation:

(2) $$\left\{\begin{array}{c} {\dot{x}}_{0}\left(t\right)=Ax_{0}\left(t\right)+Bu_{0}\left(t\right)\\ y_{0}\left(t\right)=Cx_{0}\left(t\right) \end{array}\right.$$

where $x_{0}\left(t\right)\in R^{n}$, $y_{0}\left(t\right)\in R^{m}$ is the state and output of the leader, respectively. $u_{0}\left(t\right)\in R^{p}$ is the unknown input of $x_{0}$. The leader’s signal is only available to partially following agents.

**Definition** **2.** *For the MASs described in Equations (1) and (2), if*

(3) $$\lim_{t\to \infty }∥x_{i}-d_{i}x_{0}∥=0,\;i=1,2,\ldots ,N,$$

*is satisfied, the MASs reach bipartite consensus.*

Moreover, Assumptions 1–4 and Lemma 1 are proposed to tackle the bipartite consensus issue in MASs affected by actuator faults and a leader with a nonzero input.

**Assumption** **1.** *The fault distribution matrix E is of full column rank, that is, rank(E)=q. Moreover, the following condition is satisfied for any λ where real(λ)≥0: rank $\left[\begin{array}{cc} A-\lambda I & E\\ C & 0 \end{array}\right]=n+\mathrm{rank}\left(E\right)$.*

**Assumption** **2.** *This paper takes into account the presence of actuator faults. Thus, it is assumed that $E=[B_{q_{1}},B_{q_{2}},\cdots ,B_{q_{q}}]$, where $B_{q_{j}}\left(j=1,2,\cdots ,q\right)$ is the $q_{j}$-th column of matrix B. Consequently, we know that $B\bar{E}=E$.*

**Assumption** **3.** *The fault signal $f_{i}\left(t\right)$ is subject to the condition ${\dot{f}}_{i}\left(t\right)\leq \beta_{f},\;\mathrm{where}\;\beta_{f}\geq 0$ is a constant.*

**Remark** **1.** *Assumption 1 is critical for ensuring the observability of fault signals in practical systems. This condition allows the intermediate observer to effectively separate and estimate both system states and fault signals, similar to requirements in industrial robotic systems where actuator faults must be isolated from normal operational variations. In Assumption 3, this boundedness condition reflects real-world scenarios where physical limitations prevent faults from growing indefinitely, such as in aircraft control surfaces where mechanical stops limit maximum deflection rates. In general, descriptor sliding mode observers require prior knowledge of the fault bounds and their derivatives, while the adaptive observer assumes that both faults and their first derivatives are bounded. In contrast, Assumption 3 allows that $\beta_{f}$ are unknown and that the faults are unbounded (e.g., faults are ramps), so it is more general than those used in the aforementioned methods.*

**Assumption** **4.** *$u_{0}$ is represented by a set of base functions, as shown below.*

(4) $$u_{0}=\theta_{0}^{T}\phi_{0}$$

*where $\phi_{0}$ represents the base function, and $\theta_{0}$ is a vector of constant parameters [36].*

**Remark** **2.** *In practical systems, it is inevitable for the leader to have external input. Therefore, this article designs an adaptive learning rate to estimate the unknown input signal of the leader by parameterization theory. The parameterization of $u_{0}$ is common in adaptive control (e.g., [35]). It allows the leader’s input to be reconstructed using basis functions (e.g., Fourier series), which is practical for periodic or piecewise-continuous signals.*

**Remark** **3.** *This study investigates the uncertainties in control inputs and actuator faults in multi-agent systems. For instance, in robotic teams, individual robots may experience actuator degradation or failures, leading to reduced mobility and affecting overall coordination. In autonomous UAV formations, variations in wind speed or mechanical malfunctions can result in thrust loss for certain drones, compromising formation stability. Additionally, in remotely operated unmanned systems, such as deep-sea or space exploration robots, the leader may be subject to unknown environmental disturbances, causing unpredictable input variations that affect the trajectory tracking of followers. In such scenarios, adaptive observers are essential for estimating unknown inputs and adjusting the followers’ control strategies to maintain system stability and coordination.*

**Lemma** **1** (Young’s Inequality)**.** *The following inequality can be derived for matrices X and Y:*

(5) $$XY^{T}+YX^{T}\leq cXX^{T}+\frac{1}{c}YY^{T}$$

*where c>0 is a constant.*

## 3. Main Results

### 3.1. Fault Estimation Observer Design

To design a fault estimation observer that meets performance requirement of this paper, an intermediate variable is defined as

(6) $$\zeta_{i}\left(t\right)=f_{i}\left(t\right)-Kx_{i}\left(t\right)$$

where *K* is the transition gain matrix to be designed. To simplify the expression, t is omitted below.

From (1) and (6), it can be derived that

(7) $$\dot{\zeta_{i}}=\dot{f_{i}}-K\left(Ax_{i}+Bu_{i}+E\zeta_{i}+EKx_{i}\right)$$

The faults in the MAS are estimated using the following transition variable estimator:

(8) $$\left\{\begin{array}{c} {\dot{\hat{x}}}_{i}=A{\hat{x}}_{i}+Bu_{i}+E\hat{f_{i}}+\bar{L}e_{yi}\\ {\hat{y}}_{i}=C{\hat{x}}_{i}\\ {\hat{f}}_{i}={\hat{\zeta }}_{i}+K{\hat{x}}_{i}\\ {\dot{\hat{\zeta }}}_{i}=-KE{\hat{\zeta }}_{i}-K\left(A{\hat{x}}_{i}+Bu_{i}+EK{\hat{x}}_{i}\right) \end{array}\right.$$

where ${\hat{x}}_{i},{\hat{y}}_{i},{\hat{\zeta }}_{i}$, and ${\hat{f}}_{i}$ are the estimation of $x_{i},y_{i},\zeta_{i}$ and $f_{i}$, respectively. $\bar{L}$ is the gain matrix of the estimator. $e_{yi}=y_{i}-{\hat{y}}_{i}$. Additionally, the transition gain matrix *K* is defined as

(9) $$K=\iota E^{T}$$

where ι is a scalar parameter that needs to be appropriately chosen.

**Remark** **4.** *The UIO strategy, as an excellent method for state estimation of systems, has attracted widespread attention. In recent years, several related novel methods have continuously emerged. For example, ref. [37] designs a joint UIO using an interval observer to estimate both the state of the system and unknown inputs simultaneously. In [38], a type of distributed UIO is proposed to achieve the estimation of the state of the system and unknown input. Based on the aforementioned results, this work explores the application of intermediate variable observers to the problem of bipartite fault-tolerant consensus control. Moreover, unlike most existing works on fault estimation for MASs, the proposed transition variable estimator ensures that the estimation errors converge exponentially without requiring the bounds of the faults and their derivatives.*

Defining $e_{xi}≜x_{i}-{\hat{x}}_{i},e_{fi}≜f_{i}-{\hat{f}}_{i},e_{\zeta_{i}}≜\zeta_{i}-{\hat{\zeta }}_{i}$, the error dynamics can be expressed as follows:

(10) $$\left\{\begin{array}{c} {\dot{e}}_{xi}=\left(A-\bar{L}C\right)e_{xi}+Ee_{fi}\\ {\dot{e}}_{\zeta_{i}}={\dot{f}}_{i}-\iota E^{T}Ee_{\zeta_{i}}-\iota E^{T}\left(A+\iota EE^{T}\right)e_{xi} \end{array}\right.$$

The compact form is given by

(11) $$\left\{\begin{array}{c} \begin{array}{cc} {\dot{e}}_{x} & =\left[I_{N}⊗\left(A-\bar{L}C\right)\right]e_{x}\left(t\right)+\left(I_{N}⊗E\right)e_{f}\\ {\dot{e}}_{\zeta } & =\dot{f}-\left(I_{N}⊗\iota E^{T}E\right)e_{\zeta }-\left[I_{N}⊗\left(\iota E^{T}A+\iota E^{T}\iota EE^{T}\right)\right]e_{x} \end{array} \end{array}\right.$$

where $e_{x}={\left[e_{x_{1}}^{T},\ldots ,e_{x_{N}}^{T}\right]}^{T},e_{\zeta }=[e_{\zeta_{1}}^{T}$$,\ldots ,e_{\xi_{N}}^{T}{]}^{T},e_{f}={\left[e_{f_{1}}^{T},\ldots ,e_{f_{N}}^{T}\right]}^{T},\dot{f}={\left[{\dot{f}}_{1}^{T},\ldots ,{\dot{f}}_{N}^{T}\right]}^{T}$.

**Theorem** **1.** *Under Assumptions 1–3, the proposed transition variable estimator ensures that the system’s error dynamics remain uniformly ultimately bounded. This result holds for given positive constants ι>0, β>0, and γ>0, provided that there are matrices M>0 and S>0 meeting the following criteria:*

(12) $$\Omega =\left[\begin{array}{cc} \Omega_{11} & \Omega_{12}\\ \Omega_{12}^{T} & \Omega_{22} \end{array}\right]<0$$

*where $\Omega_{11}≜A^{T}M-C^{T}S^{T}+MA-SC+\iota MEE^{T}+\iota EE^{T}M,\;\Omega_{12}≜ME-\iota \gamma A^{T}E-\iota^{2}\gamma EE^{T}E,\;\Omega_{22}≜-2\iota \gamma E^{T}E+\left(1/\beta \right)I.$*

**Proof** **of Theorem 1.** Consider the Lyapunov function defined as follows:

(13) $$V=e_{x}^{T}\left(I_{N}⊗M\right)e_{x}+e_{\zeta }^{T}\left(I_{N}⊗\Gamma^{-1}\right)e_{\zeta }$$

where $\Gamma^{-1}=\gamma I$ is a matrix to be designed.

The $\dot{V}$ is expressed as

(14) $$\begin{array}{cc} \dot{V}= & e_{x}^{T}\left\{I_{N}⊗\left[{\left(A-\bar{L}C\right)}^{T}M+M\left(A-\bar{L}C\right)\right]\right\}e_{x}\\ \; & +\;2e_{x}^{T}\left(I_{N}⊗ME\right)e_{f}-2e_{\zeta }^{T}\left(I_{N}⊗\iota \gamma E^{T}E\right)e_{\zeta }\\ \; & -\;2e_{\zeta }^{T}\left(I_{N}⊗\iota \gamma E^{T}A\right)e_{x}+2\gamma e_{\zeta }^{T}\dot{f}\\ \; & -\;2e_{\zeta }^{T}\left(I_{N}⊗\iota^{2}\gamma E^{T}EE^{T}\right)e_{x}. \end{array}$$

From (6) and (9), one can obtain that

(15) $$e_{f}=e_{\zeta }+\left(I_{N}⊗\omega E^{T}\right)e_{x}$$

Substituting (15) into (14) results in

(16) $$\begin{array}{cc} \dot{V}= & e_{x}^{T}\left\{I_{N}⊗\left[{\left(A-\bar{L}C\right)}^{T}M+M\left(A-\bar{L}C\right)\right]\right\}e_{x}\\ \; & +\;2e_{x}^{T}\left(I_{N}⊗ME\right)e_{\zeta }+2e_{x}^{T}\left(I_{N}⊗\iota MEE^{T}\right)e_{x}^{T}\\ \; & -\;2e_{\zeta }^{T}\left(I_{N}⊗\iota \gamma E^{T}E\right)e_{\zeta }-2e_{\xi }^{T}\left(I_{N}⊗\iota^{2}\gamma E^{T}EE^{T}\right)e_{x}\\ \; & -\;2e_{\zeta }^{T}\left(I_{N}⊗\iota \gamma E^{T}A\right)e_{x}+2\gamma e_{\zeta }^{T}\dot{f}. \end{array}$$

Based on Assumption 3, there are unknown scalars $\beta_{f}$ such that ${\dot{f}}_{i}\leq \beta_{f}$. Consequently,

(17) $$2\gamma e_{\zeta }^{T}\dot{f}\leq \frac{1}{\beta }e_{\zeta }^{T}e_{\zeta }+\beta \gamma^{2}\beta_{f}^{2}.$$

Thus, we have

(18) $$\begin{array}{cc} \dot{V}\leq & e_{x}^{T}\left\{I_{N}⊗\left[{\left(A-\bar{L}C\right)}^{T}M+M\left(A-\bar{L}C\right)\right]\right\}e_{x}\\ \; & +\;2e_{\zeta }^{T}\left(I_{N}⊗E^{T}M\right)e_{x}+2e_{x}^{T}\left(I_{N}⊗\iota MEE^{T}\right)e_{x}^{T}\\ \; & -\;2e_{\zeta }^{T}\left(I_{N}⊗\iota \gamma E^{T}E\right)e_{\zeta }-2e_{\zeta }^{T}\left(I_{N}⊗\iota \gamma E^{T}A\right)e_{x}\\ \; & -\;2e_{\zeta }^{T}\left(I_{N}⊗\iota^{2}\gamma E^{T}EE^{T}\right)e_{x}+\frac{1}{\beta }e_{\zeta }^{T}e_{\zeta }+\beta \gamma^{2}\beta_{f}^{2}. \end{array}$$

Denote $\tilde{e}={\left[e_{x}^{T}\;e_{\zeta }^{T}\right]}^{T}$. Then, Equation (18) can be rewritten equivalently as

(19) $$\dot{V}\leq {\tilde{e}}^{T}\Pi \tilde{e}+\beta \gamma^{2}\beta_{f}^{2}$$

where $\Pi =\left[\begin{array}{cc} \Pi_{11} & \Pi_{21}^{T}\\ \Pi_{21}^{T} & \Pi_{22} \end{array}\right]<0$, with $\Pi_{11}=I_{N}⊗\left[{\left(A-\bar{L}C\right)}^{T}M+M\left(A-\bar{L}C\right)\right]+I_{N}⊗\iota MEE^{T}+I_{N}⊗\iota EE^{T}M,\;\Pi_{21}=I_{N}⊗E^{T}M-I_{N}⊗\iota \gamma E^{T}A-I_{N}⊗\iota^{2}\gamma E^{T}EE^{T},\;\Pi_{22}=-2I_{N}⊗\iota \gamma E^{T}E+\frac{1}{\beta }.$

For (13),

(20) $$\begin{array}{cc} V & \leq \max\left[\lambda_{\max}\left(M\right),\delta \right]\left({∥e_{x}\left(t\right)∥}^{2}+{∥e_{\zeta }\left(t\right)∥}^{2}\right)\\ & =\max\left\{\lambda_{\max}\left(M\right),\delta \right\}{∥e\left(t\right)∥}^{2}. \end{array}$$

This results in the following:

(21) $${∥\tilde{e}∥}^{2}\geq \frac{V}{\max\{\lambda_{\max}\left(M\right),\delta \}}.$$

From (19), it follows that

(22) $$\dot{V}\leq \lambda_{\max}\left(\Pi \right)\cdot {∥\tilde{e}∥}^{2}+\beta \gamma^{2}\beta_{f}^{2}.$$

Obviously, $\lambda_{\max}\left(\Pi \right)<0$. Subsequently, in accordance with (21), we derive

(23) $$\lambda_{\max}\left(\Pi \right)\cdot {∥\tilde{e}∥}^{2}\leq \frac{\lambda_{\max}\left(\Pi \right)\cdot V}{\max\{\lambda_{\max}\left(M\right),\delta \}}.$$

By substituting (23) into (22), the following expression is obtained:

(24) $$\dot{V}\leq \frac{\lambda_{\max}\left(\Pi \right)}{\max\{\lambda_{\max}\left(M\right),\delta \}}V+\beta \gamma^{2}\beta_{f}^{2}.$$

Denote Ξ as follows:

(25) $$\begin{array}{cc} \Xi & =\left\{\left(e_{x},e_{\zeta }\right)|\lambda_{\max}\left(M\right)∥e_{x}{∥}^{2}+\;\delta {∥e_{\zeta }∥}^{2}\right.\\ \; & \leq -\frac{\beta \gamma^{2}\beta_{f}^{2}\cdot \max\left\{\lambda_{\max}\left(M\right),\delta \right\}}{\lambda_{\max}\left(\Pi \right)}. \end{array}$$

Thus, for $\left(e_{x},e_{\zeta }\right)\in \Xi_{s}$ (where $\Xi_{s}$ represents the complementary set of Ξ), the following inequality is satisfied.

(26) $$\begin{array}{cc} V & \geq \lambda_{\min}\left(M\right)∥e_{x}{∥}^{2}+\;\delta {∥e_{\zeta }∥}^{2}\\ \; & \geq -\frac{\beta \gamma^{2}\beta_{f}^{2}\cdot \max\left\{\lambda_{\max}\left(M\right),\delta \right\}}{\lambda_{\max}\left(\Pi \right)}. \end{array}$$

Consequently, from (24) and (26), it follows that $\dot{V}\leq 0$ holds whenever $\left(e_{x},e_{\zeta }\right)\in \Xi_{s}$.

By applying Lyapunov theory, it is shown that the pair $\left(e_{x},e_{\zeta }\right)$ is asymptotically bounded and may converge exponentially to the set Ξ at a rate exceeding $e^{\nu t}$, as described in (24), where $\nu =(\lambda_{\max}\left(\Pi \right)/\left(\max\left\{\lambda_{\max}\left(M\right),\delta \right\}\right))<0$. Additionally, leveraging the Schur complement formula, it is evident that the condition Ξ<0 is equivalent to satisfying the theorem’s condition (12) when $S=M\bar{L}$. Hence, the gain for the estimator can be expressed as $\bar{L}=M^{-1}S$. □

### 3.2. Distributed Consensus Control Protocol

The unknown inputs originating from the leader complicate the process for $x_{i}$ to precisely acquire the $x_{0}$’s information. To address this issue, a leader state observer is constructed, enabling followers to effectively estimate the leader’s state.

(27) $$\begin{array}{cc} \; & {\dot{\hat{x}}}_{0i}=A{\hat{x}}_{0i}+B{\hat{\theta }}_{i}^{T}\phi_{0}-\rho BB^{T}P_{2}\left(x_{0}-{\hat{x}}_{0i}\right) \end{array}$$

(28) $$\begin{array}{cc} \; & {\dot{\hat{\theta }}}_{i}=\tau_{1}\left(-\phi_{0}{\left(x_{0}-{\hat{x}}_{0i}\right)}^{T}P_{2}B-\alpha_{1}{\hat{\theta }}_{i}\right) \end{array}$$

where ${\hat{x}}_{0i},{\hat{\theta }}_{i}$ are the estimation of $x_{0},\theta_{0}$ by each follower agent, respectively. ρ, $\alpha_{1}$, and $\tau_{1}$ are positive constants. $P_{2}$ is a positive-definite matrix.

**Remark** **5.** *In MASs, especially in leader–follower systems, the leader’s behavior not only affects the leader’s own state but also indirectly influences the entire system’s dynamics through its input signals. In most practical scenarios, the leader’s input signals may be unobservable (e.g., due to external disturbances, noise, or unknown control signals), which prevents the followers from directly obtaining the leader’s accurate state information. Without this information, follower agents cannot properly adjust their control strategies. Therefore, to compensate for this deficiency and ensure the stable and reliable operation of the multi-agent system, we have designed a leader state observer. This observer can estimate the leader’s state based on the known system dynamics and the leader’s output signals, enabling the followers to achieve effective coordination and control even without directly receiving the leader’s state.*

However, the existence of actuator faults combined with the unknown input signals from the leader poses significant challenges in analyzing bipartite consensus problems.

(29) $$\begin{array}{cc} \; & u_{i}=-c_{i}K_{1}\varphi_{i}-\bar{E}{\hat{f}}_{i}+d_{i}{\hat{\theta }}_{i}^{T}\phi_{0}\\ \; & {\dot{c}}_{i}=\tau_{2}\left(\varphi_{i}^{T}P_{1}BB^{T}P_{1}\varphi_{i}\right) \end{array}$$

where $K_{1}$ represents the gain matrix that will be determined later, $c_{i}$ is the dynamic gain, and its minimum value is $c_{n}$. $\varphi_{i}$ represents the relative observer error:

(30) $$\varphi_{i}=\sum_{j=1}^{N}\left|a_{ij}\right|\left({\hat{x}}_{i}-\mathrm{sgn}\left(a_{ij}\right){\hat{x}}_{j}\right)+g_{i}\left({\hat{x}}_{i}-d_{i}{\hat{x}}_{0i}\right)$$

Then, the bipartite observer error and the bipartite observer consensus error are defined as follows:

(31) $$r_{i}=x_{i}-d_{i}{\hat{x}}_{0i}$$

(32) $${\hat{r}}_{i}={\hat{x}}_{i}-d_{i}{\hat{x}}_{0i}$$

Define $e_{x_{0i}}=\left(x_{0}-{\hat{x}}_{0i}\right)$, and the bipartite consensus error as $\delta_{i}=x_{i}-d_{i}x_{0}$. It can be observed that, if $\lim_{t\to \infty }e_{x_{0i}}=0$ and $\lim_{t\to \infty }r_{i}=0$, $\lim_{t\to \infty }r_{i}=0$, indicating that the MASs achieve bipartite consensus.

According to (1) and (27), it can be shown that

(33) $$\begin{array}{cc} {\dot{r}}_{i} & ={\dot{x}}_{i}-d_{i}{\dot{\hat{x}}}_{x_{0i}}\\ \; & =Ar_{i}+Bu_{i}+Ef_{i}-d_{i}\left(B{\hat{\theta }}_{i}^{T}\phi_{0}-\rho BB^{T}P_{2}e_{x_{0i}}\right)\\ \; & =Ar_{i}-c_{i}BK_{1}\varphi_{i}+d_{i}\rho BB^{T}P_{2}e_{x_{0i}}-Ee_{fi}. \end{array}$$

Let $r={\left[r_{1}^{T},r_{2}^{T},\ldots ,r_{N}^{T}\right]}^{T},e_{x0}={\left[e_{x01}^{T},e_{x02}^{T},\ldots ,e_{x0N}^{T}\right]}^{T}$. The global error dynamic is given by

(34) $$\begin{array}{cc} \dot{r}= & \left(I_{N}⊗A\right)r-\left(I_{N}⊗cBK_{1}\right)\left(L_{G}⊗I_{N}\right)\hat{r}\\ \; & +\rho \left(D⊗BB^{T}P_{2}\right)e_{x_{0i}}-\left(I_{N}⊗E\right)e_{f}. \end{array}$$

Since ${\hat{r}}_{i}=r_{i}-e_{x_{i}}$, substituting it into (34) yields

(35) $$\begin{array}{cc} \dot{r}= & \left(I_{N}⊗A\right)r-\left(L_{G}⊗cBK_{1}\right)r+\rho \left(D⊗BB^{T}P_{2}\right)e_{x_{0}}\\ \; & -\left(H⊗BK_{1}\right)e_{x}-\left(I_{N}⊗E\right)e_{f}. \end{array}$$

Given that $e_{x}$ and $e_{f}$ can converge under the conditions of Theorem 1, Equation (35) can be rewritten in the following form as t→∞:

(36) $$\dot{r}=\left(I_{N}⊗A\right)r-\left(L_{G}⊗cBK_{1}\right)r+\rho \left(D⊗BB^{T}P_{2}\right)e_{x_{0}}.$$

Similarly, it can be obtained that

(37) $$\begin{array}{cc} {\dot{e}}_{x_{0i}} & ={\dot{\hat{x}}}_{0}-{\dot{\hat{x}}}_{0i}\\ \; & =Ax_{0}+Bu_{0}-A{\hat{x}}_{0i}-B{\hat{\theta }}_{i}^{T}\phi_{0}+\rho BB^{T}P_{2}e_{x_{0i}}\\ \; & =Ae_{x_{0i}}+B{\tilde{\theta }}_{i}^{T}\phi_{0}+\rho BB^{T}P_{2}e_{x_{0i}}. \end{array}$$

where $\tilde{\theta_{i}}=\theta_{i}-{\hat{\theta }}_{i}$.

The compact form is given by

(38) $$\begin{array}{c} {\dot{e}}_{x_{0}}=\left(I_{N}⊗A\right)e_{x_{0}}+\left(I_{N}⊗B\right){\tilde{\theta }}^{T}\Phi_{0}+\rho \left(I_{N}⊗BB^{T}P_{2}\right)e_{x_{0}}. \end{array}$$

**Remark** **6.** *Theorem 2 establishes sufficient conditions for achieving bipartite consensus under actuator faults. The key requirements are as follows: (1) The existence of positive definite matrices P1 and P2 satisfying the matrix inequalities (39) and (40). (2) The feedback gain matrix is given by $K_{1}=\varrho B^{T}P_{1}$, where $\varrho =Re{\left(\lambda_{i}\right)}^{-1}$. This formulation guarantees the convergence of the control law and ensures the stability of the closed-loop system. (3) The estimation errors $e_{x0}$ and $e_{fi}$ converge to zero within a finite time, ensuring that followers accurately estimate both the leader’s state and fault signals, thereby maintaining overall system stability. These conditions ensure that the Lyapunov function decreases over time, guaranteeing system stability and consensus achievement.*

**Theorem** **2.** *Assuming that Assumptions 1–4 hold, and the feedback gain matrix meets $K_{1}=\varrho B^{T}P_{1}$, where $\varrho =Re{\left(\lambda_{i}\right)}^{-1}$, and $\lambda_{i}$ represents the eigenvalue of $L_{G}$. There are positive constants $c_{n}$, $\beta_{1}$, and a symmetric positive-definite matrix $P_{1}$, $P_{2}$, and $W_{1}$, which satisfy the following conditions:*

(39) $$\begin{array}{cc} \; & P_{1}A+A^{T}P_{1}-2c_{n}BB^{T}+\frac{1}{\beta_{1}}P_{1}BW_{1}B^{T}P_{1}<0 \end{array}$$

(40) $$\begin{array}{cc} \; & P_{2}A+A^{T}P_{2}+\beta_{1}P_{2}BW_{1}^{-1}B^{T}P_{2}+2\rho P_{2}BB^{T}P_{2}<0 \end{array}$$

*The bipartite consensus control is guaranteed under signed digraphs with actuator faults.*

**Proof** **of Theorem 2.** The Lyapunov function candidate is considered as follows:

(41) $$\bar{V}=r^{T}\left(I_{N}⊗P_{1}\right)r+e_{x_{0}}^{T}\left(I_{N}⊗P_{2}\right)e_{x_{0}}+\sum_{i=1}^{N}\frac{1}{\tau_{1}}{\tilde{\theta_{i}}}^{T}\tilde{\theta_{i}}$$

From (36) and (38), the derivative of $\bar{V}$ is

(42) $$\begin{array}{cc} \dot{\bar{V}}= & 2r^{T}\left(I_{N}⊗P_{1}A\right)r-2r^{T}\left(L_{G}⊗cP_{1}BK_{1}\right)r\\ \; & +\;2r^{T}\left(D⊗\rho P_{1}BB^{T}P_{2}\right)e_{x_{0}}+2e_{x_{0}}^{T}\left(I_{N}⊗P_{2}A\right)e_{x_{0}}\\ \; & +\;2e_{x_{0}}^{T}\left(I_{N}⊗P_{2}B\right){\tilde{\theta }}^{T}\Phi_{0}+2\rho e_{x_{0}}^{T}\left(I_{N}⊗P_{2}BB^{T}P_{2}\right)e_{x_{0}}\\ \; & +\;2\sum_{i=1}^{N}{\tilde{\theta }}_{i}^{T}\left(-\phi_{0}e_{x_{0i}}^{T}P_{2}B\right)+2\sum_{i=1}^{N}{\tilde{\theta }}_{i}^{T}\left(-\alpha_{1}{\hat{\theta }}_{i}\right). \end{array}$$

It is true that

(43) $$2e_{x_{0}}^{T}\left(I_{N}⊗P_{2}B\right){\tilde{\theta }}^{T}\Phi_{0}=2\sum_{i=1}^{N}\left(e_{x_{0i}}^{T}P_{2}B{\tilde{\theta }}_{i}^{T}\phi_{0}\right)$$

(44) $$-{\tilde{\theta }}^{T}{\hat{\theta }}_{i}=-{\tilde{\theta }}_{i}^{T}\left({\tilde{\theta }}_{i}+\theta_{0}\right)\leq \frac{1}{4}\theta_{0}^{2}.$$

Consequently, one can obtain that

(45) $$\begin{array}{cc} \dot{\bar{V}}= & 2r^{T}\left(I_{N}⊗P_{1}A\right)r-2r^{T}\left(L_{G}⊗cP_{1}BK_{1}\right)r\\ \; & +\;2\rho e_{x_{0}}^{T}\left(I_{N}⊗P_{2}BB^{T}P_{2}\right)e_{x_{0}}+2e_{x_{0}}^{T}\left(I_{N}⊗P_{2}A\right)e_{x_{0}}\\ \; & +\;2r^{T}\left(D⊗\rho PP_{1}BB^{T}P_{2}\right)e_{x_{0}}+\sum_{i=1}^{N}\frac{\alpha_{1}}{2}\theta_{0}^{2}. \end{array}$$

By applying Lemma 1, it follows that

(46) $$\begin{array}{cc} \begin{array}{c} r^{T}\left(D⊗\rho P_{1}BB^{T}P_{2}\right)e_{x_{0}}\leq \end{array}\\ \; & \begin{array}{c} \frac{1}{\beta_{1}}r^{T}\left(I_{N}⊗P_{1}B\right)W_{1}{\left(I_{N}⊗P_{1}B\right)}^{T}r \end{array}\\ \; & +\;\beta_{1}e_{x_{0}}^{T}{\left(D⊗\rho B^{T}P_{2}\right)}^{T}W_{1}^{-1}\left(D⊗\rho B^{T}P_{2}\right)e_{x_{0}} \end{array}$$

Hence, (45) can be rewritten as

(47) $$\begin{array}{cc} \dot{\bar{V}}\leq & r^{T}[I_{N}⊗\left(P_{1}A+A^{T}P_{1}\right)-2L_{G}⊗c_{n}P_{1}BK_{1}\\ & +\;\frac{1}{\beta_{1}}\left(I_{N}⊗P_{1}B\right)W_{1}{\left(I_{N}⊗P_{1}B\right)}^{T}]r+e_{x_{0}}^{T}[I_{N}⊗\left(P_{2}A+A^{T}P_{2}\right)\\ & +\;\beta_{1}{\left(D⊗\rho B^{T}P_{2}\right)}^{T}W_{1}^{-1}\left(D⊗\rho B^{T}P_{2}\right)+2I_{N}⊗\rho P_{2}BB^{T}P2]e_{x_{0}}\\ & +\;\frac{\alpha_{1}}{2}\theta_{0}^{2}. \end{array}$$

Since $K_{1}=\varrho B^{T}P_{1}$, by choosing $P_{1}$ with condition (39), the following equation is satisfied:

(48) $$\begin{array}{cc} A_{1}= & I_{N}⊗\left(P_{1}A+A^{T}P_{1}\right)-2H⊗c_{n}P_{1}BK_{1}\\ & +\;\frac{1}{\beta_{1}}\left(I_{N}⊗P_{1}B\right)W_{1}{\left(I_{N}⊗P_{1}B\right)}^{T}<0 \end{array}$$

Similarly, selecting $P_{2}$ in combination with condition (40) yields

(49) $$\begin{array}{cc} A_{2}= & I_{N}⊗\left(P_{2}A+A^{T}P_{2}\right)+\beta_{1}{\left(D⊗\rho B^{T}P_{2}\right)}^{T}W_{1}^{-1}\left(D⊗\rho B^{T}P_{2}\right)\\ & +\;I_{N}⊗\left(\rho P_{2}BB^{T}P2+\rho P_{2}BB^{T}P_{2}\right)<0 \end{array}$$

Let $\bar{X}={\left[r^{T}\;e_{x_{0}}^{T}\right]}^{T}$. According to (47), (48), (49), and the inequality condition in Theorem 2, it follows that

(50) $$\dot{\bar{V}}\leq {\bar{X}}^{T}\Theta \bar{X}+\epsilon$$

where $\epsilon =\frac{\alpha_{1}}{2}\theta_{0}^{2},\Theta =\mathrm{diag}\left\{A_{1},A_{2}\right\}$.

According to (50),

(51) $$\dot{\bar{V}}\leq -\beta_{2}{∥\bar{X}∥}^{2}+\epsilon$$

Let $\beta_{2}=\lambda_{\min}\left(-\Theta \right)$, which represents the smallest eigenvalue of −Θ. It can then be shown that, for every $∥\bar{X}∥\;\geq \sqrt{\frac{\epsilon }{\beta_{2}}}$, $\dot{\bar{V}}\leq 0$. Consequently, the consensus error asymptotically converges to a region bounded by a sphere with a radius of $\sqrt{\frac{\epsilon }{\beta_{2}}}$. □

**Remark** **7.** *Existing studies [16,17,18,19] focus on achieving bipartite consensus but overlook the impact of actuator faults, limiting their applicability in real-world scenarios where faults are inevitable. In contrast, our work integrates fault-tolerant control into bipartite consensus, ensuring robust performance under actuator failures. While [26,27] consider bipartite fault-tolerant consensus, their approaches are restricted to undirected graphs or assume a leader with no input, which constrains practical implementation. Our method extends to directed signed graphs and effectively handles unknown leader inputs, making it more versatile. Additionally, works such as [29,30,31,32,33] impose bounded leader input assumptions, which may not always hold in practice. By employing a parameterization-based observer, our approach relaxes this constraint, significantly enhancing applicability and robustness in dynamic environments.*

## 4. Simulation Results

**Figure 1:**
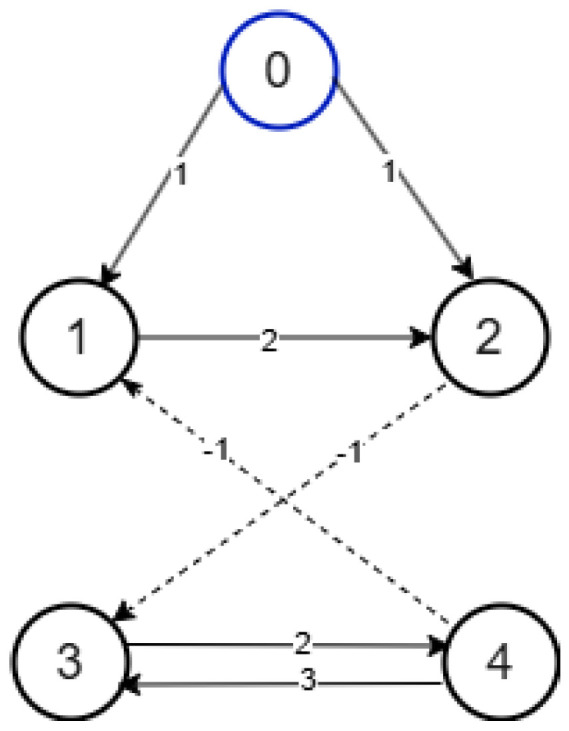
Topology of the MASs.

This section presents a simulation example to illustrate the effectiveness of the proposed approach. Based on Equations (1) and (2), the simulation parameters are specified as follows. In this numerical example, we analyze a MAS with four agents. The structure of the MAS is shown in Figure 1.

As shown in Figure 1, the adjacency matrix A and the Laplacian matrix are given as follows: L of the MAS are derived as follows.

$$A=\left[\begin{array}{cccc} 0 & 2 & 0 & 0\\ 0 & 0 & -1 & 0\\ 0 & 0 & 0 & 2\\ -1 & 0 & 3 & 0 \end{array}\right],L=\left[\begin{array}{cccc} 2 & -2 & 0 & 0\\ 0 & -1 & 1 & 0\\ 0 & 0 & 2 & -2\\ 1 & 0 & -3 & 2 \end{array}\right]$$

Clearly, as we consider a directed graph, the Laplacian matrix L is not symmetric.

This study considers the effect of actuator faults. The system parameters for the *i*-th agent (i=1,2,3,4) are defined as follows:

$$A=\left[\begin{array}{ccc} 1 & 2 & \\ -2 & 1 & \end{array}\right],B=E=\left[\begin{array}{c} 1\\ 1 \end{array}\right],C=\left[\begin{array}{ccc} 1 & 1 & \\ 0 & 1 & \end{array}\right]$$

**Remark** **8.** *The system parameters A, B, C, and E define the dynamics of each agent in the MAS: A represents the inherent dynamics of an individual agent, determining its state evolution in the absence of external inputs. B defines how control inputs influence the agent’s state, shaping its ability to follow desired trajectories. C specifies the output variables available for feedback, affecting the observability and estimation of agent states. E characterizes how actuator faults impact the system, ensuring that faults can be identified and mitigated through proper estimation. These parameters collectively dictate how agents interact, respond to inputs, and adapt to disturbances, ultimately shaping the overall system behavior and its ability to achieve bipartite consensus under faults.*

The nodes are grouped into two sets: $V_{p}=\left\{1,2\right\}$ and $V_{q}=\left\{3,4\right\}$. The leader’s unknown input signal is specified as $u_{0}=0.7\sin\left(t\right)$. To assess the performance of the proposed protocols, actuator faults are introduced at different time intervals for each agent. The faults for the agents are described as follows:

$$\begin{array}{cc} f_{1}\left(t\right) & =\sin(0.2t)\\ f_{2}\left(t\right) & =\left\{\begin{array}{c} 0,\;0s⩽t<10s\\ \cos(0.1t-1)+\sin(0.3t-3)-1,\;t\geq 10s \end{array}\right.\\ f_{3}\left(t\right) & =\left\{\begin{array}{c} 0,\;0s\leq t<15s\\ 2(1-e^{-0.5t+7.5}),\;15s\leq t<70s\\ 2\left[1-(1-e^{-0.5t+35})\right],\;t\geq 70s \end{array}\right.\\ f_{4}\left(t\right) & =\left\{\begin{array}{c} 0,\;0s\leq t<15s\\ \cos(0.2t-3+\frac{\pi }{2}).\;t\geq 15s \end{array}\right. \end{array}$$

Using the fault estimator described in the previous section, the constant ω is chosen as 6. According to Theorems 1 and 2, the other parameters $\bar{L}$, $P_{1}$ and $P_{2}$ can be calculated.

$$\bar{L}=\left[\begin{array}{cc} 60.2346 & 3.6715\\ -21.2365 & 30.5379 \end{array}\right],P_{1}=\left[\begin{array}{cc} 5.3733 & -0.3662\\ -0.3662 & 0.9438 \end{array}\right],P_{2}=\left[\begin{array}{cc} -4.5655 & 0.7925\\ 0.7925 & -1.9544 \end{array}\right]$$

**Remark** **9.** *The parameter values were selected through systematic tuning to optimize observer performance. For instance, ω=6 was chosen through numerical optimization to balance estimation speed and stability, while $P_{1}$ and $P_{2}$ were computed using LMI techniques to satisfy the stability conditions in Theorems 1 and 2. The observer gain matrix L was derived from these solutions to ensure robust fault estimation.*

**Figure 2:**
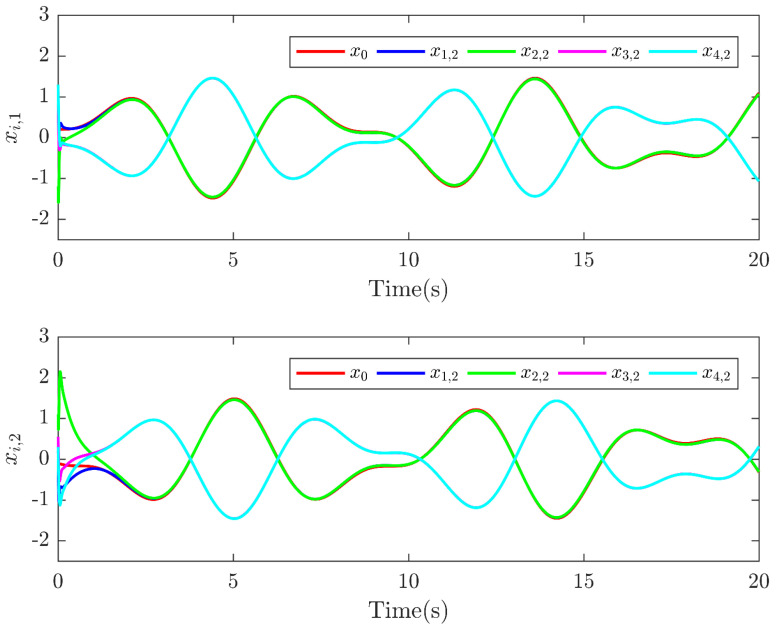
Trajectories of $x_{0}$ and $x_{i}$.

**Figure 3:**
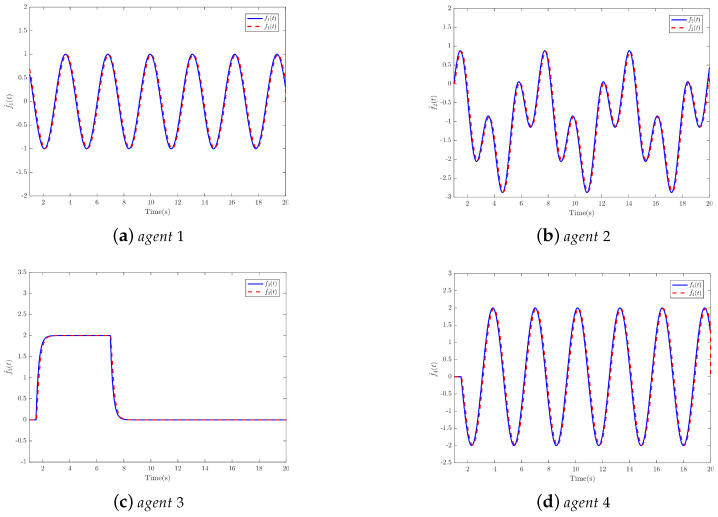
Faults and its estimations.

**Figure 4:**
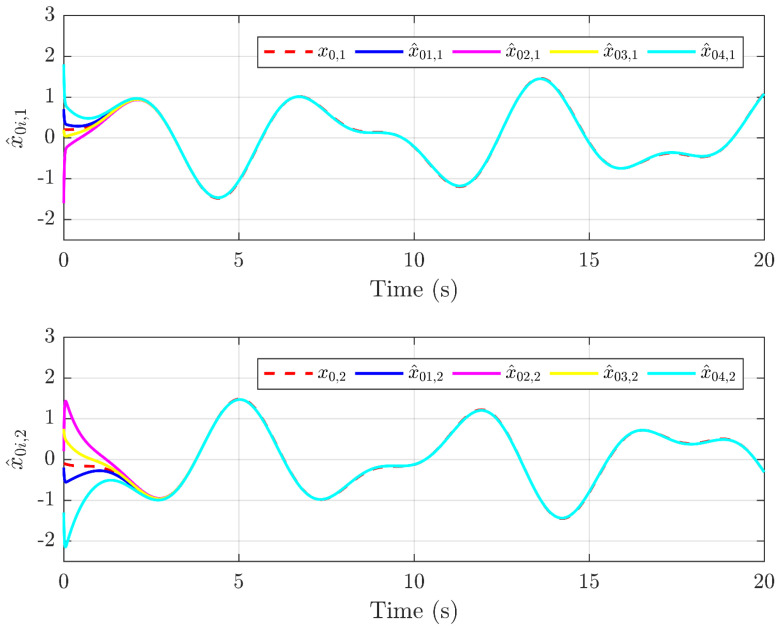
Trajectories of the leader $x_{0}$ and observers ${\hat{x}}_{0i}$.

**Figure 5:**
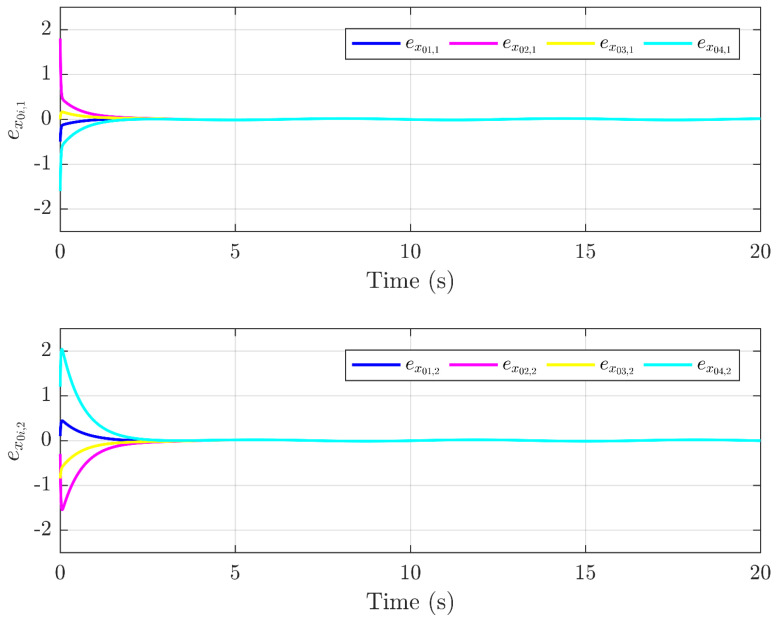
Trajectories of the error $e_{xoi}$.

**Figure 6:**
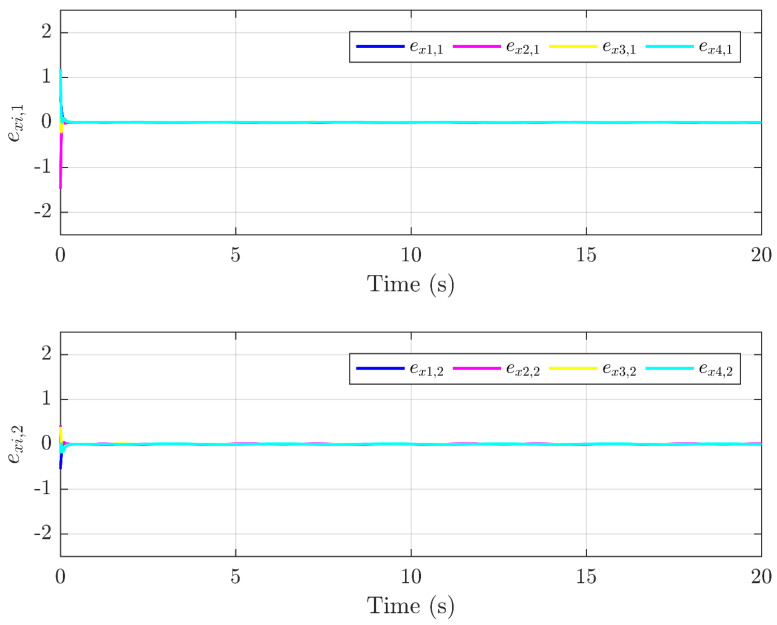
Trajectories of the error $e_{xi}$.

**Figure 7:**
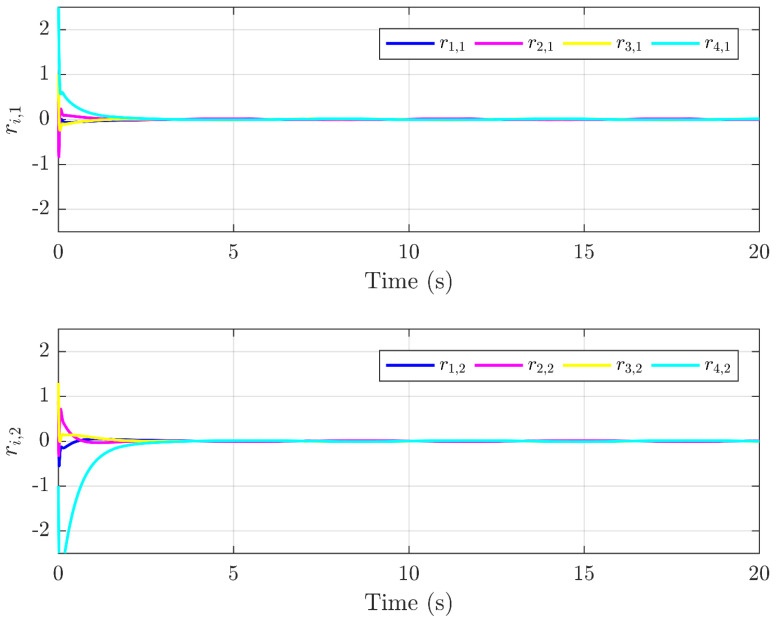
Trajectories of the error $r_{i}$.

Next, the simulation results are provided in Figure 2, Figure 3, Figure 4, Figure 5, Figure 6 and Figure 7. Figure 2 illustrates the trajectories of the leader and follower agents, demonstrating that the proposed control strategy enables the followers to track the leader while achieving bipartite consensus. Figure 3 compares actual actuator faults with their estimated values, verifying that the designed fault estimation observer accurately identifies faults in real time. Figure 4 shows the leader state estimation errors, highlighting that the adaptive observer effectively tracks the leader’s state despite unknown input disturbances. Figure 5 presents the observer error dynamics, confirming the convergence of the intermediate observer and the accuracy of state estimation. Figure 6 depicts the state estimation errors of the follower agents, showing that the proposed observer design ensures precise state tracking. Figure 7 displays the bipartite consensus errors, which converge to zero over time, validating that the proposed control protocol successfully achieves bipartite consensus under actuator faults. Collectively, these results confirm the robustness and effectiveness of the proposed method in ensuring fault-tolerant bipartite consensus, even in the presence of unknown leader inputs and actuator faults.

As shown in Figure 7, the bipartite consensus error $r_{i}$ converges to zero within t = 25 s, demonstrating rapid stabilization despite actuator faults. Figure 3 reveals that the proposed observer achieves a mean absolute error of 0.04 for $f_{3}\left(t\right)$ and 0.05 for $f_{2}\left(t\right)$, validating high estimation precision even under abrupt fault changes. The protocol maintains stability when faults with varying magnitudes (e.g., $f_{3}\left(t\right)$ transitions from 0 to 2 s) are introduced, as evidenced by the bounded error in Figure 5.

## 5. Conclusions

This paper introduces a new distributed strategy for bipartite fault-tolerant consensus control in MASs under signed directed graphs, addressing the challenges of actuator faults and unknown leader input signals. Compared to existing methods, the proposed approach (a) enhances adaptability to cooperative-competitive multi-agent networks by considering signed directed topologies, (b) improves robustness, addressing both actuator faults and unknown leader inputs through intermediate and adaptive observers, and (c) broadens applicability, eliminating the need for strict observer matching conditions, making it suitable for diverse real-world scenarios. The study integrates intermediate observers and adaptive estimators to precisely evaluate faults and leader states. Theoretical analysis using Lyapunov stability demonstrates that the proposed approach ensures bipartite consensus, even under actuator faults and varying input conditions. Simulation results validate the robustness and feasibility of the control strategy, confirming its potential for practical applications in systems requiring reliable and fault-resilient multi-agent coordination. Future research will explore stochastic disturbances to further enhance the adaptability of the proposed control framework. Additionally, practical validation in autonomous vehicle fleets, smart grids, and robotic systems will be conducted to assess its real-world applicability.

## Data Availability

No new data were created.
